# Syndrome de choc toxique staphylococcique chez un hémodialysé chronique

**DOI:** 10.11604/pamj.2017.27.230.12737

**Published:** 2017-07-28

**Authors:** Hassan Alaoui, Ayoub Belhadj, Younes Aissaoui, Rachid Seddiki, Mohamed Zoubir, Mohamed Bougalem

**Affiliations:** 1 Pole d’Anesthésie-Réanimation, Hôpital Militaire Avicenne, Faculté de Médecine 40000 Marrakech, Maroc

**Keywords:** Syndrome de choc toxique staphylococcique, staphylocoque doré, hémodialyse, fistule artério-veineuse, Staphylococcal toxic shock syndrome, staphylococcus aureus, hemodialysis, arteriovenous fistula

## Abstract

Le syndrome de choc toxique staphylococcique est un syndrome infectieux aigu et systémique lié à l'activité super-antigénique des toxines staphylococciques. C'est une pathologie qui est assez rare mais reste grevée d'une mortalité considérable malgré la prise en charge thérapeutique. La porte d'entrée est le plus souvent cutanée avec dissémination bactériémique secondaire pourrait être sujet de mesures préventives. Nous rapportons le cas d'un choc toxique à staphylocoque doré d'évolution rapidement mortelle, développé chez un hémodialysé chronique dont la porte d'entrée à partir de la fistule artério-veineuse était soupçonnée.

## Abstract (en)

Staphylococcal toxic shock syndrome is an acute and systemic infectious syndrome associated with the super-antigenic activity of staphylococcal toxins. It is a pathology that is rather rare but remains burdened with a considerable mortality despite the therapeutic management. The gateway is usually cutaneous with secondary bacteremic spread could be subject to preventive measures. We report the case of a rapidly fatal staphylococcal toxic shock, developed in a chronic hemodialysis whose entry from the arteriovenous fistula was suspected.

## Introduction

Le syndrome de choc toxique staphylococcique (SCT) est un syndrome infectieux aigu causé par les toxines du staphylocoque aureus, c'est une complication infectieuse systémique rare, avec une incidence annuelle en France de 1/100 000, mais grave car potentiellement létale [1]. Il est classique d'opposer les SCT d'origine menstruelle, ayant une mortalité assez faible (< 5%), qui survient lors de l’utilisation de tampons vaginaux « superabsorbants », à ceux d'origine non menstruelle, survenant au décours d’une infection suppurative et dont la mortalité avoisine les 20 % [1]. Nous rapportons le cas d'un patient âgé ayant une néphropathie diabétique au stade de dialyse admis pour une pneumopathie communautaire grave ayant précédé un SCT staphylococcique dont l'évolution était malheureusement mortelle.

## Patient et observation

**Figure 1:**
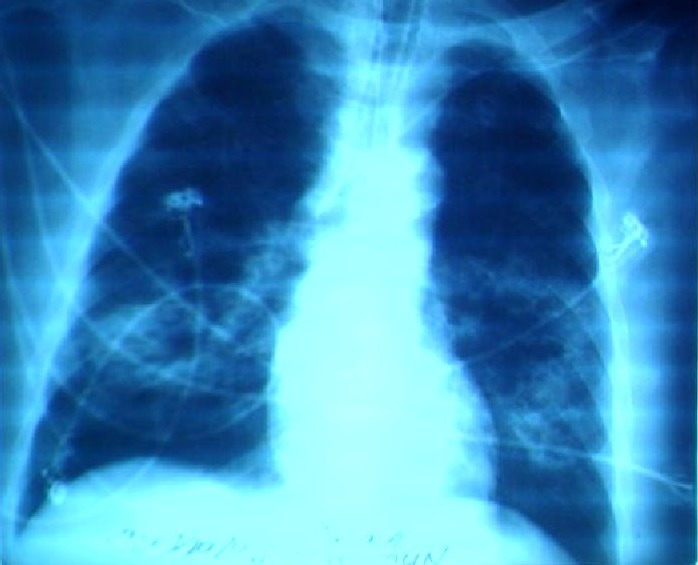
Aspect de pneumopathie bilatérale

**Figure 2:**
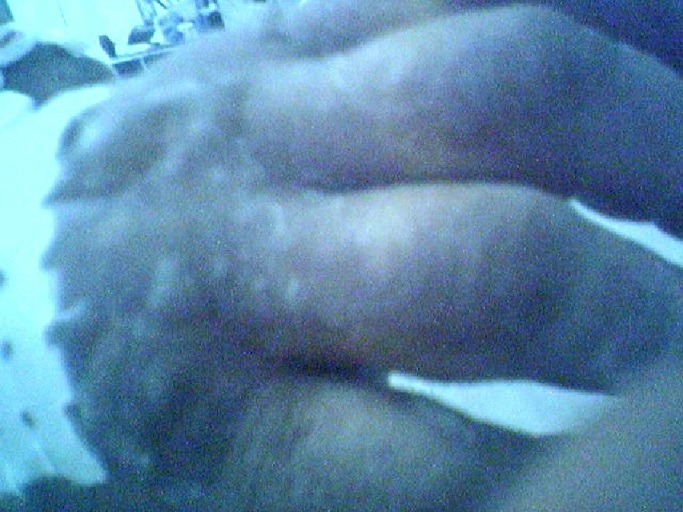
Aspect de dermatose bulleuse au niveau des mains

Il s'agit d'un patient âgé de 73 ans connu diabétique et hémodialysé chronique, admis aux urgences pour une détresse respiratoire aigue faite d'une polypnée à 32c/mn, des sueurs profuses et une SpO_2_ = 88% à l'air ambiant, avec une fièvre à 39.5, une tachycardie à 123b/mn, une PAS à 125mmHg et PAD à 74mmHg. Après mise en conditionnement, un bilan sanguin complet était demandé avec une gazométrie artérielle, des lactates et des hémocultures. Une radiographie pulmonaire était réalisée et a objectivé une pneumopathie bilatérale (Figure 1), le bilan sanguin a montré des GB à 38000, une glycémie à 2.97g/l sans acétone, des lactates à 4.18 et une hypoxémie à 65mmHg avec une capnie normale sous oxygène, l'index d'oxygénation était à 225. Une antibiothérapie initiale à base de pénicilline protégé et quinolone était introduite. Ensuite, le malade a présenté une instabilité hémodynamique avec PAS à 92mmHg et PAD à 57mmHg, avec une désaturation et des troubles de conscience, ce qui a obligé d'intuber et ventiler le patient, mettre une voie veineuse centrale et une ligne artérielle pour monitorer la volémie et la pression artérielle, puis la malade était transféré en réanimation où il a déclaré son choc septique. L'évolution était marquée par la suite par l'aggravation radiologique et gazométrique à J1 avec un SDRA, qui a coïncidé avec l'apparition d'une éruption cutanée au niveau du tronc avec une dermatose bulleuse et desquamative au niveau des deux mains (Figure 2) dont les prélèvements ont isolé un staphylocoque aureus méticilline sensible. Le même germe était retrouvé également sur les prélèvements distaux protégés et les hémocultures, tandis que le diagnostic d'endocardite infectieuse était éliminé par l'échodoppler cardiaque. Notre patient est décédé à J3 à la suite de son choc toxique rebelle avec défaillance multiviscérale.

## Discussion

Le syndrome du choc toxique (SCT) stapylococcique est une maladie infectieuse rare et aigue, dont la 1^ère^description remonte en 1978. Elle est potentiellement létale et provoqué par la diffusion dans l'organisme de la toxine TSST-1 (toxic shock syndrome toxin-1) et/ou des entérotoxines [1]. Cette toxine TSST-1, est un des nombreux facteurs de virulence associé au staphylocoque aureus, ou staphylocoque doré. Elle fait partie de la famille des superantigènes capables d'induire une activation polyclonale des lymphocytes T. Ceci a pour conséquence une libération massive de cytokines (TNF, IL1 et 6), une sensibilisation des monocytes aux endotoxines et des lésions de l'endothélium expliquant la survenue d'hypotension et du choc staphylococcique (fièvre, hypotension, hémorragie) [1, 2]. Le diagnostic positif reste avant tout clinique et repose sur l'association d'une hyperthermie, d'une instabilité hémodynamique ou d'un état de choc, d'une érythrodermie scarlatiniforme plus ou moins desquamative généralisée ou palmoplantaire et d'une atteinte multisystémique d'organes en effet des critères de définition ont été établis par le center for disease control and prevention (Tableau 1) [3]. La porte d´entrée ou l'origine de l'infection peut être cutanée (coupure, brûlure, incision chirurgicale), vaginale (tampon hygiénique) ou ORL (sinusite, pharyngite), comme elle peut passer complètement inaperçue. La mise en évidence du staphylocoque aureus sur les différents prélèvements bactériologiques est rare et son isolement au niveau de lésions cutanées et/ou sur les hémocultures suffit pour confirmer le diagnostic [2, 3]. Parmi les facteurs de risques rapportés on trouve la présence de lésions cutanéo-muqueuses même minimes, l'antibiothérapie antérieure à large spectre, l'immunodépression acquise ou congénitale avec absence d'anticorps anti TSST-1 [4] et enfin les affections chroniques (diabète, muscoviscidose, cancer, insuffisance rénale chronique). Dans le cas de notre patient diabétique et hémodialysé chronique, le SCT pourrait être à point de départ hématique au niveau de la fistule artério-veineuse avec dissémination bactériémique staphylococcique objectivée sur les hémocultures. La coexistence de la pneumopathie pourrait être expliquée aussi par la dissémination bactériémique à partir de la fistule, comme elle pourrait être elle même à l'origine du SCT. La thérapeutique anti-SCT repose sur des mesures conventionnelles de réanimation visant l'état de choc et la défaillance multiviscérale, associées à une antibiothérapie active contre le staphylocoque aureus, de type béta-lactamines. L'utilité d'y associer un antibiotique susceptible d'inhiber la synthèse protéique bactérienne tel que la clindamycine est suggérée par une étude in vivo et reste licite [5]. La place des traitements immunomodulateurs tels que les immunoglobulines polyvalentes reste discutée, mais ils sont préconisés par de nombreuses équipes [6].

**Tableau 1:** Syndrome du choc toxique: critères diagnostiques

Critères majeurs	Atteinte multi systémique (au moins 3 critères)
Température > 38.9◦C	Gastro-intestinal : vomissements ou diarrhée au début
Érythrodermie maculaire diffuse ± Desquamation à prédominance palmo-plantaire survenant 1 à 2 semaines après le début du SCT	Musculaire: myalgies sévères ou élévation de la CPK à au moins 2 fois la normale
	Muqueuse: hyperémie vaginale, oro-pharyngée ou conjonctivale
	Rénale: urée ou créatinine sanguine à au moins 2 fois la normale
TA systolique ≤ 90 mmHg chez l’adulte	Hépatique: bilirubine totale ou ASAT-ALAT à au moins 2 fois la normale
	Neurologique: désorientation ou altération de la conscience sans déficit neurologique focal

## Conclusion

Bien que rare, le SCT reste une pathologie grave et potentiellement mortelle dont la prise en charge idéale doit être ciblée aussi sur la prévention et le traitement de toutes les lésions cutanées staphylococciques comme étant des portes d'entrée éventuelles.

## Conflits d’intérêts

Les auteurs ne déclarent aucun conflits d'intérêts.
